# A Case of Strangulated Crural Hernia, Where the Operation Succeeded after the Obstruction Had Continued Six Days

**Published:** 1801-02

**Authors:** G. Borrett

**Affiliations:** Surgeon, Great Yarmouth, and Member of the Royal College of Surgeons, London.


### *A Case of strangulated Crural Hernia, where the Operation succeeded after the Obstruction had continued Six Days.*

By G. Borrett, Surgeon, Great Yarmouth, and Member of the Royal College of Surgeons, London.

---

### *To the Editors of the Medical and Phyfical Journal.*

Gentlemen,

On Tuefday, Sept. 25, 1800, I was requefted to attend Mrs. Warner, of this place, in confultation with four medical gentlemen. We found her with a *Crural Hernia* incarcerated, vomiting a quantity of fæcal matter, with hiccough, cold extremities, and a quick, fmall, but irregular pulfe.

The inteftine had already been ftrangulated five days, during which time the ufual treatment recommended for reduction was fteadily but unfuccefsfully purfued. The tumour was about the fize of a pullet's egg; and in confequence of its not being tender to the touch, reduction was attempted, but without fuc-

---

cefs. The operation appeared the only chance to fave her life, which was urged to her, but fhe would not confent. The cold applications were continued, pills compofed of Ext. Colocynth. c. and Calomel and a grain of Opium for a dofe, were prefcribed, and enemas of tobacco. At ten o'clock the following morning we vifited our patient. The enemas had acted powerfully on the fyftem, but produced no evacuation by ftool; the vomiting of fæces continued. Being apprehenfive that fhe could live very few hours, fhe begged the operation might be performed, to which we agreed, as the only means of faving her life; although we now much doubted its fuccefs, as her pulfe was become very feeble, and fhe had the *facies hippocratica* ftrongly marked.

Every neceffary preparation being made, I proceeded to the operation; expofed the fascia, ligamentum Poupartii, the hernial fac, and its contents; I then divided the ligamentum Poupartii, and afterwards, with fome difficulty, paffed Pott's Biftory, and divided the ftricture (which was confiderable) at the mouth of the fac. The rupture was Enterocele, and the portion of inteftine included, which was the fize of a nutmeg, appeared of a livid colour: Preffure upon the inteftine gave pain and excited retchings, which encouraged me to hope it had ftill fufficient life to have its action reproduced.

The fac had defcended for fourteen years, and more than once fuffered inflammation; it was thickened in confequence, and refembled a cod's found, and completely adhered to the furrounding adipofe membrane; a great portion of it was diffected out. The wound was drawn together by futures; light dreffings were applied, and fecured by a T bandage. Small opiates were ordered every four hours, to allay the irritability of the ftomach, and fupport her ftrength; and broth clyfters, after fome days, reftored the natural periftaltic motion of the inteftines. Mrs. Warner was the mother of thirteen children, and at this time in the third month of geftation; fhe did not mifcarry, and is now quite well: She wears a trufs. This cafe, and many others recorded, fhew, that the operation for Hernia may fucceed at almoft any period of ftrangulation, and encourage us never to defpair of our patient.

I am induced to communicate the above cafe, becaufe Mr. Geoghegan, (No. xx. of your ufeful Medical Journal for October) in his "Strictures on the ufual Practice in ftrangulated Hernia," reprefents the danger of the operation, abftractedly confidered, to be imminent, and appears an advocate for unlimited procraftination; from which, in my eftimation, the danger wholly arifes, and becaufe I know of no clear account of the operation for *Crural Hernia.*

Whoever

Whoever will confult Pott on Ruptures, will fee the moft judicious treatment and decided reafons for his mode of conduct, refpecting the operation. "There is no circumftance," fays he, "attending ruptures with ftricture, in which more variety is found than in the time which they will fafely admit to be fpent in their reduction; fome have been fuccefsfully replaced at the end of eight or ten days, others have proved fatal in one." The attention of the medical world ought, therefore, to be directed to the *time* when the operation, in different cafes of ftrangulated Hernia, fhould be performed; from delay every thing is to be dreaded; and many valuable lives are daily loft, that would indifputably be faved. The Surgeon who has a cafe of ftrangulated Hernia, ought not to wafte the only moments which might fecure his patient's life by a *well-timed operation* in applying fomentations and placebos. Pott, p. 82 and 83, vol. ii. "There is not, in the practice of Surgery, a point which requires more judgment, firmnefs or delicacy, than to determine the precife time beyond which this operation fhould not be deferred, and for a furgeon to conduct himfelf fo as to induce a patient to fubmit to it early enough for his prefervation. The two principal circumftances which have moft contributed to the infrequency of performing this operation are, a dread of great hazard from the operation itfelf, confidered abftractedly, and a fear of bringing a difgrace upon it by having performed it too late; *ne occidisse, nisi servasset videretur.**— The firft then is vaftly greater than it ought to be, and is moft frequently the caufe of the latter; fo that if the one can juftly be leffened, the other will not be likely to happen." Few experienced men then can doubt the propriety of operating early in ftrangulated Hernia: After the moft approved treatment has been regularly and confiftently tried without effect, and a judicious application of the taxis has been unfuccefsful, why fhould the life of the patient be rifqued by farther delay of the operation, in which, comparatively, there is no hazard, if it be judicioufly performed? I am juftified in this pofitive affertion if any inference may be drawn from fix cafes, four † of which (befides the prefent) recovered. Of the other two, one died from the operation being too long delayed; and the failure of the other depended on the ftricture at the mouth of the fac not having been completely divided; a point in the operation which requires great care and certainty.

* Celfus.

† From one of thefe patients more than a pound of difeafed omentum was cut off, without hæmorrhage or any other difagreeable fymptom.

I entirely agree with Mr. Geoghegan in the application of cold to the tumour, by cloths wetted in mixtures generating cold, as well as by expofing the body to cold, where the patient can bear it; but I don't fee how the palms of the hands fhould be applied to the fides of the Hernia denominated by writers Bubonocele, which is the term of the difeafe of which Mr. G. treats: In Scrotal Hernia indeed they may; but while the contents of the rupture are fituated in the groin, it appears impoffible that the palms of the hands fhould be applied in the manner Mr. G. recommends, to expel air. The quotation which he makes from Mr. Pott, relative to grafping the tumour, is not only inaccurate, but the application is erroneous, it being in *Scrotal Hernia* Mr. Pott recommends that the tumour fhould be grafped with one hand, to prevent the teftes from afcending and the inteftine from defcending; while, with the other hand, you endeavour the return of the tumour. Had Mr. G. read a little farther, he would have feen the following. "If the cafe be a Bubonocele, there will be no occafion for endeavouring to grafp the tumour; but by continued moderate preffure on it with the fingers, to endeavour the return of the piece of gut." Neither am I inclined to confider the abdominal ring as a metal ring, which therefore "muft be paffive;" is it not more probable, that the diforder in the inteftine is originally produced by the ftricture made upon it by the borders of the tendinous opening of the abdominal mufcle, and that the gut is in general perfectly found and free from difeafe before it becomes engaged in fuch ftricture, and that the inflammation of the inteftine is the confequence? If the abdominal ring is paffive, how will Mr. G. explain the frequent reduction of ruptures during the relaxation and fyncope occafioned by the tobacco enema, and the ftill more frequent reduction which takes place a few hours before death, from thefe very parts having loft their tone, and no longer making refiftance to the return of the Hernia?*

In the operation for Crural Hernia, the epigaftric artery and fpermatic chord in the male, and ligamentum rotundum in the female, are parts lying in the way of the knife, and particularly increafe the difficulty and hazard of the fuccefs of this operation. Benjamin Bell, in his account of the operation in Femoral Hernia, difcourages us by faying, "Whether the incifion be carried directly upwards, or even obliquely outwards or

* Since the above Cafe, I am informed a female has died in Yarmouth of ftrangulated Crural Hernia, to whom medical affiftance was not called till fhe was in articulo mortis; when upon applying the fingers to the tumour, a crepitus was heard, and the contents went up: The patient died foon after.

or inwards, thefe parts muft generally be divided." Pott is not more clear and determined, and recommends, after the hernial fac, inteftine, and ligamentum Poupartii, &c. are expofed, to endeavour the return of the inteftine before the divifion of the ligament. Suppofing every medical man perfectly acquainted with the anatomy of the parts concerned in Crural Hernia, I fhall proceed directly to a defcription of the operation.

The patient being placed in a proper fituation, the incifion is to be begun above the tumour, laying bare the fafcia and the fac. You then fee the ligamentum Poupartii, binding down the fac, which is to be divided; but as you will have the epigaftric artery, and fpermatic veffels in man, croffing each other *immediately over* the tumour; and in woman the epigaftric artery and ligamentum rotundum, much care muft be taken.—The beft mode is, to make a fmall incifion between the fibres of the external oblique mufcle, about *half an inch* above the ligament, and a director being paffed *carefully and immediately under the ligament, and over the artery*, (which adheres to the ligament by cellular fubftance) to cut into the grove of the director. If, when this is done, you fhould find the ftricture at the mouth of the fac, it muft be divided *inwards* to the pubes, inclining Pott's knife rather obliquely downwards. In this mode you will avoid the artery, chord, and round ligament.

I conclude with apologies for taking up fo much of your Journal; but if, through your means, the attention of the Medical World fhould be directed to an early operation in thefe dangerous cafes, we fhall be fufficiently rewarded by the many lives which will be faved. I am, &c.

